# Development and content validation of the Long COVID/ post-acute sequelae of COVID-19 (PASC) patient-reported outcome (PRO) instrument

**DOI:** 10.1186/s41687-025-00942-w

**Published:** 2025-08-20

**Authors:** Dale Chandler, Benjamin Abramoff, Candace Bramson, Joseph. C. Cappelleri, Aishwarya Chohan, Magdalena Harrington, Hiba Jamal, Jillian Lusk, Iyar Mazar, Roger Paredes, Sophi Tatlock, Andrew Ustianowski, Edward Weinstein, Ruth Mokgokong

**Affiliations:** 1 Patient-Centered Outcomes, Adelphi Values Ltd, Bollington, UK; 2 University of Pennsylvania, Philadelphia, USA; 3 Pfizer Inc, New York, USA; 4 Department of Infectious Diseases, Hospital Germans Trias i Pujol and irsiCaixa, Badalona, Spain; 5 Regional Infectious Diseases Unit, North Manchester General Hospital, Manchester, UK; 6 Pfizer Ltd, Tadworth, UK

**Keywords:** Cognitive debriefing (CD), Concept elicitation (CE), Content validity, Patient-reported outcome (PRO), Qualitative interviews, Long COVID, Post-acute sequelae of COVID-19

## Abstract

**Background:**

Post-acute sequelae of COVID-19 (PASC) or Long COVID is a post-viral complication of SARS-CoV-2 infection, causing ongoing symptoms and impaired function over a prolonged period of time. There is limited understanding of how the signs and symptoms of Long COVID/PASC affect patients’ lives and how to measure them, which is essential when developing care strategies. This study aimed to develop and evaluate the content validity for a novel patient-reported outcome (PRO) instrument in Long COVID/PASC through qualitative research, which was informed by the patient experience of Long COVID/PASC and both patient and clinician input.

**Methods:**

A review of literature and PRO instruments developed for Long COVID/PASC identified measurement gaps for the context of use (i.e., weekly assessment of signs/symptoms in clinical trial research). This informed the development of the preliminary Long COVID/PASC PRO instrument, which was tested via patient interviews (combined concept elicitation and cognitive debriefing) to align with regulatory standards, and discussions with clinical experts were conducted to provide clinical insights. The final instrument was modified based on this input to further promote its content validity.

**Results:**

Thirty participants were interviewed about their Long COVID/PASC experiences. Participants most frequently reported experiencing tiredness after physical activity (*n* = 29/30; 97%), general tiredness (*n* = 28/30; 93%), shortness of breath (*n* = 25/30; 83%), cough (*n* = 23/30; 77%) and muscle/body aches (*n* = 23/30; 77%). All participants reported that Long COVID/PASC had an impact on their health-related quality of life. Almost all (*n* = 27/28; 96%) sign/symptom concepts were reported in the first three sets of interviews suggesting conceptual saturation was achieved. Items, response options and the recall period of the preliminary Long COVID/PASC PRO instrument were understood as intended (≥ 90%) and relevant to most participants across both rounds (≥ 47%). Modifications were made to the instrument following patient input, resulting in the 18-item Long COVID/PASC instrument. Clinician input (*n* = 3) corroborated participant interview results, supporting the content validity of the Long COVID/PASC PRO instrument.

**Conclusion:**

The Long COVID/PASC PRO instrument has been developed in line with regulatory standards and the qualitative evidence demonstrated strong content validity in a Long COVID/PASC population. Research to evaluate psychometric properties will provide further evidence of the instrument’s measurement properties.

**Supplementary Information:**

The online version contains supplementary material available at 10.1186/s41687-025-00942-w.

## Introduction

Coronavirus Disease 2019 (COVID-19) is caused by severe acute respiratory syndrome coronavirus 2 (SARS-CoV-2). Patients in the acute phase present with a range of possible signs and symptoms including respiratory (cough, dyspnea, expectoration, and chest pain), constitutional (fever, fatigue/tiredness and chills/shivers), rheumatic (muscle and joint pain), otolaryngological (sore throat, taste disorder, loss of smell, rhinorrhea), gastrointestinal (loss of appetite, diarrhea, nausea or vomiting) and neurological (confusion or brain fog). The severity of acute symptoms varies significantly [1–4].

Post-acute sequelae of COVID-19 (PASC), or Long COVID, is a complication of SARS-CoV-2 infection, whereby patients continue to experience symptoms beyond the acute phase of their COVID-19 infection (i.e., starting from at least 4 to 12 weeks since initial diagnosis, and lasting at least 6 weeks or longer) [5–7]. This is important as recent evidence has estimated that the condition may be present in 10 to ≥ 35% of SARS-CoV-2-infected, with approximately 7% of US adults currently experiencing Long COVID [6]. There is no global consensus on the definition of Long COVID/PASC in both research and clinical settings [8]. Various agencies have generated their own definitions, including the World Health Organization (WHO; through an international Delphi consensus process) [9], the UK National Institute for Health and Care Excellence (NICE) [7], the US Centers for Disease Control and Prevention (CDC) [10], and, recently, the US National Academies of Sciences, Engineering and Medicine [6]. Although ‘Long COVID’ is widely used by researchers and preferred by patients, it does not yet have a consistent definition.

There is limited published qualitative research regarding the patient experience of Long COVID/PASC; however, early reports indicate that residual effects of SARS-CoV-2 infection can include fatigue/tiredness, brain fog, shortness of breath, body aches, gastrointestinal symptoms, cardiovascular symptoms and decline in quality of life [11–14]. At present, there is no approved pharmacological treatment or preventive therapy for Long COVID/PASC. The paucity of robust, patient-centred research limits the understanding of how Long COVID affects daily functioning and overall quality of life, which is crucial for informing care strategies and regulatory decision-making.

Patient-reported outcome (PRO) instruments are being increasingly incorporated into clinical trials to measure outcomes, as recommended by regulators during patient-focused drug development (PFDD), alongside traditional clinical endpoints, to capture the patient’s perspective regarding their signs, symptoms, and daily functioning [15–18]. The FDA recommends that PRO development should involve qualitative research with the target patient population to identify the most relevant disease-related concerns, which should be reflected in the instrument’s items [15, 16]. As there are various definitions of Long COVID/PASC, this study contributes to the current body of knowledge through the development of a tool to understand the signs/symptoms from the patient perspective.

A review of existing PRO measures developed in Long COVID/PASC, and a targeted literature review were conducted. These activities identified that no measures were appropriate to use in Long COVID/PASC, based on conceptual comprehensiveness and given the intended context of use which was to use a PRO instrument via weekly administration.

The aims of this research were to conduct qualitative research in adults with a physician-confirmed diagnosis of Long COVID/PASC, and clinicians caring for patients with Long COVID/PASC to (1) identify and describe the signs, symptoms, and health-related quality of life (HRQoL) impacts of the disease and (2) evaluate the content validity of a novel instrument to assess the key signs and symptoms of Long COVID/PASC.

## Methods

### Instrument development

A targeted review of literature and instruments used in Long COVID/PASC published between 2020 and 2023 (peer-reviewed publications, conference abstracts, patient forums and existing COVID-19 instruments) informed development of an initial Long COVID/PASC conceptual model. The evidence review determined that existing instruments were not suitable for the assessment of Long COVID/PASC in a clinical trial setting: the instruments did not assess symptom severity, or would be burdensome due to a large number of items or unsuitable recall period (see supplemental materials for the conceptual model and evidence review summary). Thus, it was determined that a new instrument to measure the symptoms of Long COVID/PASC should be developed.

A draft 17-item instrument was developed in line with best practice guidance (e.g., from the FDA and COSMIN criteria), using findings from the evidence review and the initial conceptual model, to measure the key signs and symptoms of Long COVID/PASC [16, 19]. Response options were concept-specific, designed to assess frequency or severity of the symptom on a 3-to-5-point verbal response scale (VRS) to align with the existing COVID-19 signs and symptoms diary [20]. As Long COVID/PASC is considered to be the development, or continuation, of COVID-19 symptoms over an extended period of time, 7 days was deemed an appropriate recall period to capture potential changes in the symptom experience but also limit risk of recall bias [21, 22]. The draft instrument was developed in a paper-based format to simulate how it would look on an eCOA device.

### Study design

**Fig. 1:**
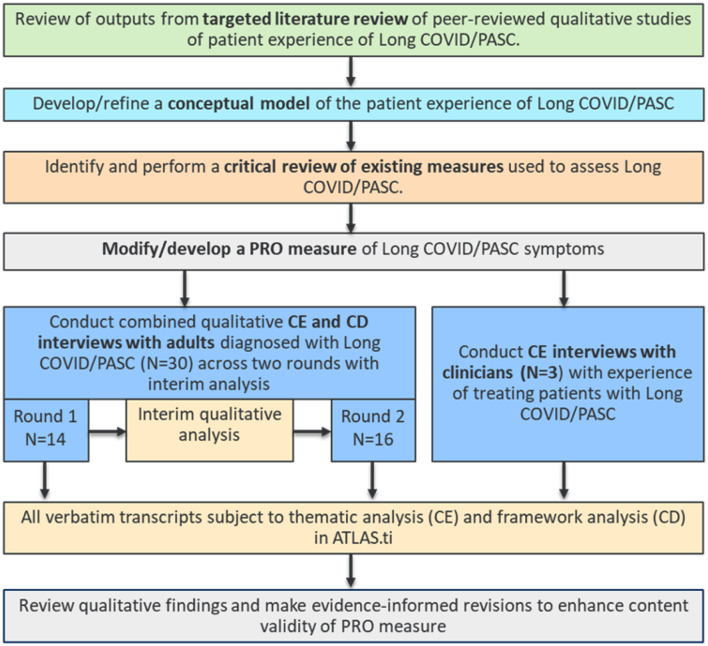
Overview of study design. Abbreviations: CE = Concept elicitation; CD = Cognitive debriefing; FDA = Food and Drug Administration; PASC = Post-acute sequelae SARS-CoV-2; PRO = Patient-reported outcome

The study employed a staged methodological approach (Fig. 1) towards PRO instrument development, aligning with regulatory guidance and best measurement practice [15, 16, 19, 23]. The qualitative interviews conducted as part of this study were cross-sectional and non-interventional. Semi-structured combined concept elicitation (CE) and cognitive debriefing (CD) interviews were conducted with US participants to inform the development of the Long COVID/PASC PRO instrument and provide evidence of content validity. Interviews were conducted in two rounds to facilitate the conduct of interim analysis and subsequent testing of modifications made to the measure following round 1 interviews. Clinicians were also interviewed to confirm clinical relevance of the patient experience of Long COVID/PASC. Ethical approval for this study was overseen by Western Copernicus Group Independent Review Board (WCG IRB; IRB tracking number: 20232556). All participants and clinicians provided oral and written informed consent prior to participating in any study-related activities.

### Study population

Participants were recruited by MedQuest Global (Valencia, CA), a third-party recruitment agency, via referring clinicians (primary care and respiratory specialists) from six geographically diverse locations within the USA (Chicago, Illinois; Baltimore, Maryland; Los Angeles, California; St. Louis, Missouri; Pittsburgh, Pennsylvania; Minneapolis, Minnesota). Age, sex, race, ethnicity and education sampling targets were applied to the sample population to ensure a range of demographic characteristics. The target sample size was determined by the principles of conceptual saturation, which is defined as the point at which no new concepts relevant to a condition is identified with the repeated collection of data [24–26]. Saturation can be achieved in as few as 12 interviews, thus it was estimated that a total sample size of 30 participants (*n* ~ 15 for each round of interviews) would be adequate to achieve conceptual saturation for each round and ensure sufficient demographic diversity.

Adult participants ≥ 18 years of age who met WHO criteria [9] for confirmed COVID-19 infection (a positive Nucleic Acid Amplification Test (NAAT) or Rapid Antigen Test) and were currently experiencing signs and/or symptoms due to COVID-19 (confirmed by their physician) were eligible to participate. The symptoms should have been present on day 30 after their Long COVID/PASC diagnosis or started after day 30 and lasted for at least 60 days. The signs and/or symptoms may have been persistent, recurrent or may have developed after the initial infection. The patient should also have experienced two post-COVID-19 signs and/or symptoms of moderate or severe intensity, as reported by their clinician within a recruitment screener, and be willing to report all (or absence of) COVID-19 vaccinations. Detailed eligibility criteria are included in the supplemental materials.

Three clinicians were also recruited for interviews. To be eligible, these clinicians had to be practicing for at least 10 years (this criterion was relaxed if the clinician evidenced considerable experience working with Long COVID/PASC patients), consulting with/managing at least four adult Long COVID/PASC patients per month, and consulting with/managing Long COVID/PASC for at least a year. All clinicians were identified via the authors’ networks.

All participants and clinicians were compensated for their participation.

### Qualitative interviews

Both rounds of combined CE and CD interviews were conducted by trained interviewers via telephone. Interviews were performed in two rounds to facilitate the conduct of interim analysis and testing of modifications made to the Long COVID/PASC PRO instrument following round 1 interviews. Each participant interview lasted approximately 90 min, with breaks offered as needed. Interviews were conducted one-on-one. The CE portion of the participant interviews began with open-ended questions to allow for spontaneous elicitation of concepts from participants regarding their experiences of Long COVID/PASC. This facilitated the exploration of signs, symptoms, and impacts experienced, and the language that participants used to describe these concepts in an unbiased manner. For example, interviewers asked participants to describe their experience of Long COVID/PASC. To capture the spectrum of relevant symptoms, interviewers used focused prompts, in participant-friendly language, to explore symptoms that were not spontaneously mentioned. This ensured that potentially applicable signs/symptoms (as identified in the evidence review) were fully explored.

Following the CE section, the CD portion of the interviews assessed the content validity of the Long COVID/PASC PRO instrument. A paper-based version of the instrument was used for the interviews. During the CD portion of the interview, participants were asked to complete the Long COVID/PASC PRO instrument and provide feedback as part of a “think aloud” exercise in which they were asked to read and complete each item, providing any thoughts or opinions about the items as they did so. Participants were also asked detailed questions about item relevance, item wording, instructions, recall period, response options, and ease of completing the items.

Following the first round of interviews, feedback from the CE portion determined if modifications to the conceptual model and Long COVID/PASC PRO instrument were needed. Based on feedback from the CD portion of the interviews, participant understanding of the instructions, items, response options, and recall periods in the instrument was assessed and wording was modified for clarity. Additionally, item relevance and overlap among items was assessed to determine if items should be added or deleted prior to the second round of interviews.

Qualitative clinician CE interviews were conducted via telephone to generate supportive evidence of the patient experience of Long COVID/PASC from a clinical perspective. Clinician interviews lasted approximately 60 min and were conducted one-to-one. Broad, open-ended questions were first used to prompt spontaneous responses, followed by more focused questions to probe on topics of interest. Clinicians were then asked to review the conceptual model of Long COVID/PASC which was drafted following the evidence review, and were questioned about the clinical relevance and comprehensiveness of the concepts within the model.

### Data analysis

Demographic and clinical characteristics of participants and clinicians were tabulated using descriptive statistics. All interviews were audio-recorded and transcribed verbatim. Qualitative analysis of participant and clinician interview transcripts was conducted using ATLAS.ti software (Scientific Software Development GmbH B, Germany). The CE sections of transcripts were analyzed using thematic analysis. Participant quotes related to experiences of Long COVID/PASC were assigned corresponding concept codes, with additional coding to indicate whether the responses were spontaneous or elicited through probing. The CD sections of transcripts were analyzed using a framework approach.

Dichotomous codes were assigned to each item, instruction, response option(s), and recall period to indicate relevance, understanding, or difficulty to complete. Codes were also used to indicate why each response option was chosen and how the content of each item applied to the patient experience, as well as to provide suggestions for item, instruction, or response option wording or formatting changes, and general feedback. Data relating to the meaningful change of concepts was also coded for, including what level of improvement or worsening was meaningful. An induction-abduction approach was taken to identify themes in the data [27].

Conceptual saturation analysis of the participant CE data was conducted, where responses from each interview were grouped by concept and the first spontaneous mention of each concept was highlighted. If no new concepts were identified in the final set of interviews, then saturation was considered to have occurred.

Exploratory sub-analyses of participant interview data were conducted to compare findings between high-risk and low-risk participants diagnosed with Long COVID/PASC. The high-risk group was defined as participants who were 50 + years old, and/or had a comorbid condition (i.e., asthma, diabetes, heart conditions), and/or were unvaccinated, in line with CDC resources and classification [28]; remaining participants were considered low-risk.

## Results

### Demographic and clinical characteristics

Thirty participants were interviewed (14 in round 1; 16 in round 2 between June and September 2023). The mean age of the sample was 42.2 years (SD = 17.4; range 20–80 years), with a greater proportion of females (*n* = 19/30; 63%) than males (*n* = 11/30; 37%). Most participants were white (*n* = 17/30; 57%); however, a range of racial and ethinic groups was represented. The sample comprised of diverse educational backgrounds, with just over half having completed college education or above (*n* = 16/30; 53%) and the remaining having completed high school or lower (*n* = 14/30; 47%).

On average, signs and/or symptoms persisted from, or developed after, the initial COVID-19 infection for 2.8 months (SD = 2.5; range = 1–13 months) prior to Long COVID/PASC diagnosis. Additionally, on average, participants had been diagnosed with Long COVID/PASC symptoms for 92.4 days (SD = 12.7; range = 70–120 days). Most participants were vaccinated (*n* = 25/30, 83%). One participant was hospitalized due to their COVID-19 and received corticosteroids as treatment. Most participants had a known infection with COVID-19 once (*n* = 19/30, 63%), and the remaining eleven participants (37%) were infected twice. The sample was further divided into a high-risk group and a low-risk group of developing severe COVID-19 for exploratory analyses [28]. More than half the sample were classified as high-risk participants (*n* = 18/30; 60%), the remainder were classed as low-risk (*n* = 12/30; 40%).

Three clinicians were interviewed; each had at least eight years of experience as a practicing clinician with at least two years of experience working with patients with Long COVID/PASC. All clinicians had papers published on Long COVID/PASC and COVID-19 and two clinicians are/were involved in Long COVID/PASC and COVID-19 clinical trials.

See the supplementary materials for detailed reporting of the demographic and clinical characteristics.

### Participant concept elicitation

**Fig. 2:**
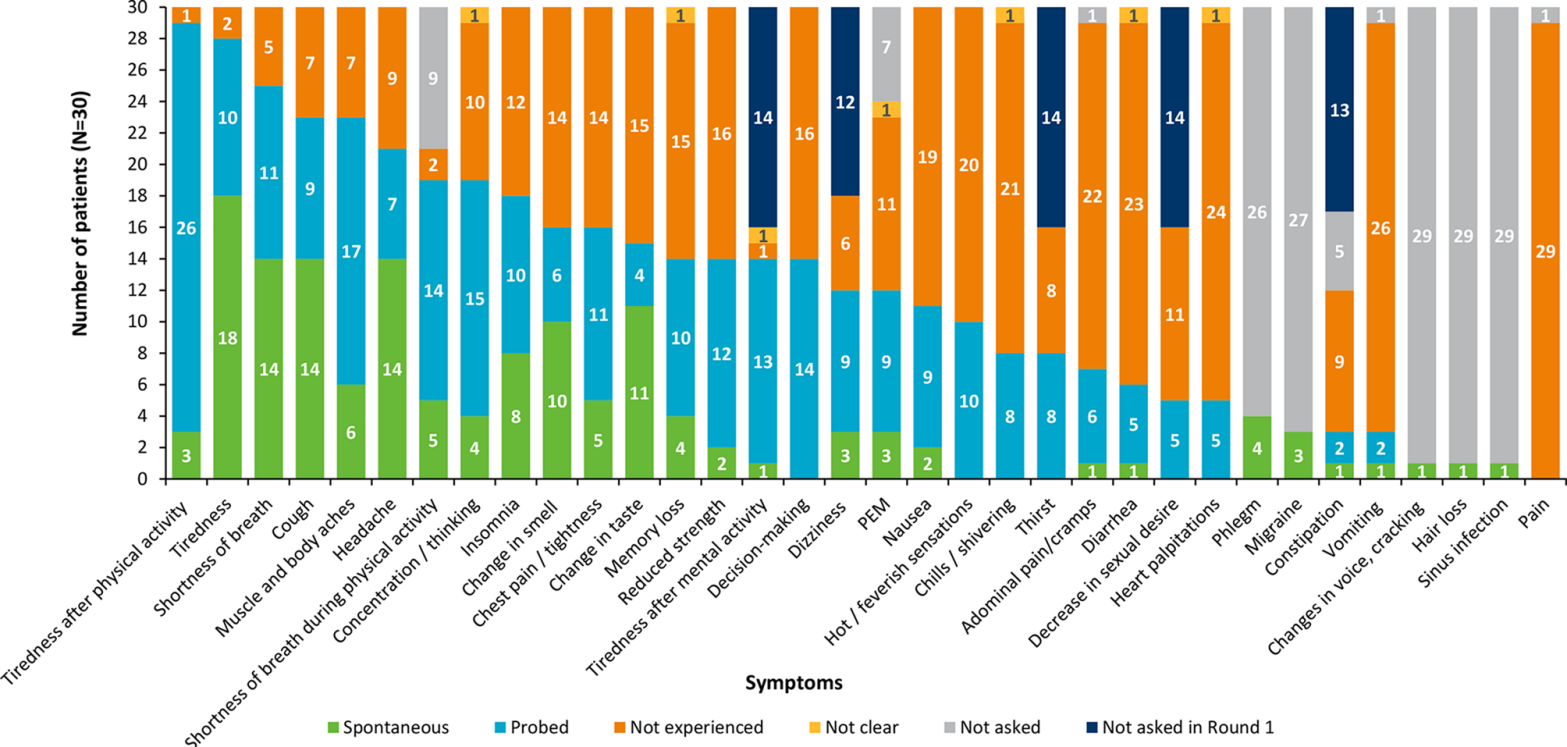
Frequencies of patient-reported signs and symptoms (*N*=30). Note: The ‘not asked’ counts for dizziness, phlegm, migraine, constipation, sinus infection, hair loss and changes in voice/cracking reflect the fact that they were additional signs and symptoms reported spontaneously by participants outside the main concepts of interest. As such, the interviewer did not ask the full sample whether they experienced these symptoms. PEM (post-exertional malaise) refers to participants who reported a worsening of signs or symptoms, other than tiredness, after physical and/or mental activity (as defined by the CDC). Counts are not intended to quantify prevalence, but instead aim to illustrate the relevance and salience of reported concepts

Thirty-four signs and/or symptoms were reported (28 spontaneously) by participants (N = 30; Fig. 2); most frequently reported symptoms (> n = 10/30; 33%) were tiredness after physical activity (n = 29/30; 97%), general tiredness (n = 28/30; 93%), shortness of breath (n = 25/30; 83%), cough (n = 23/30; 77%), muscle/body aches (n = 23/30; 77%), headache (n = 21/30; 70%), shortness of breath after physical activity (n = 19/30; 63%), difficulty concentrating or thinking (n = 19/30; 63%), and insomnia (n = 18/30; 60%). Examples of how the most frequently reported signs and/or symptoms were described by individual participants include:

“*Sometimes like if I’m doing something outside or maybe like I’m doing*,* you know*,* up and down the stairs doing the laundry*,* I get a little*,* uh*,* where I have to like stop and rest cause I’m-I’m tired.”* (Participant 17).

“*It just feels like the wind is knocked out of me. Like I’m struggling to breathe*.” (Participant 2).

*“I mean*,* I*,* sometimes feel like my throat is a little dry and I have like a dry cough.”* (Participant 4).

Participants were asked to rate how severe a sign or symptom was from 0 to 10, where 0 is not severe at all and 10 is the most severe they could imagine. Signs and symptoms were considered most salient in terms of self-reported severity score, if reported by > 50% participants with an average severity score > 4.2 (thresholds were not pre-defined or based on formal analyses, but were used as potential indicators of severity). These included: headache (6.5), tiredness after physical activity (5.7), general tiredness (5.5), shortness of breath at rest (5.1) and cough (4.3).

Participants rated how bothersome a symptom was from 0 to 10, where 0 is not bothersome at all and 10 is the most bothersome they could imagine. Signs and symptoms were considered salient in terms of their bothersomeness score if reported by ≥ 50% participants and with an average bothersomeness score of > 5.1 (thresholds were not pre-defined or based on formal analyses, but were used as potential indicators of bothersomeness). These included: general tiredness (7.0), headache (6.8), shortness of breath at rest (6.5), tiredness after physical activity (5.9), muscle and body aches (5.4) and cough (5.2).

**Fig. 3:**
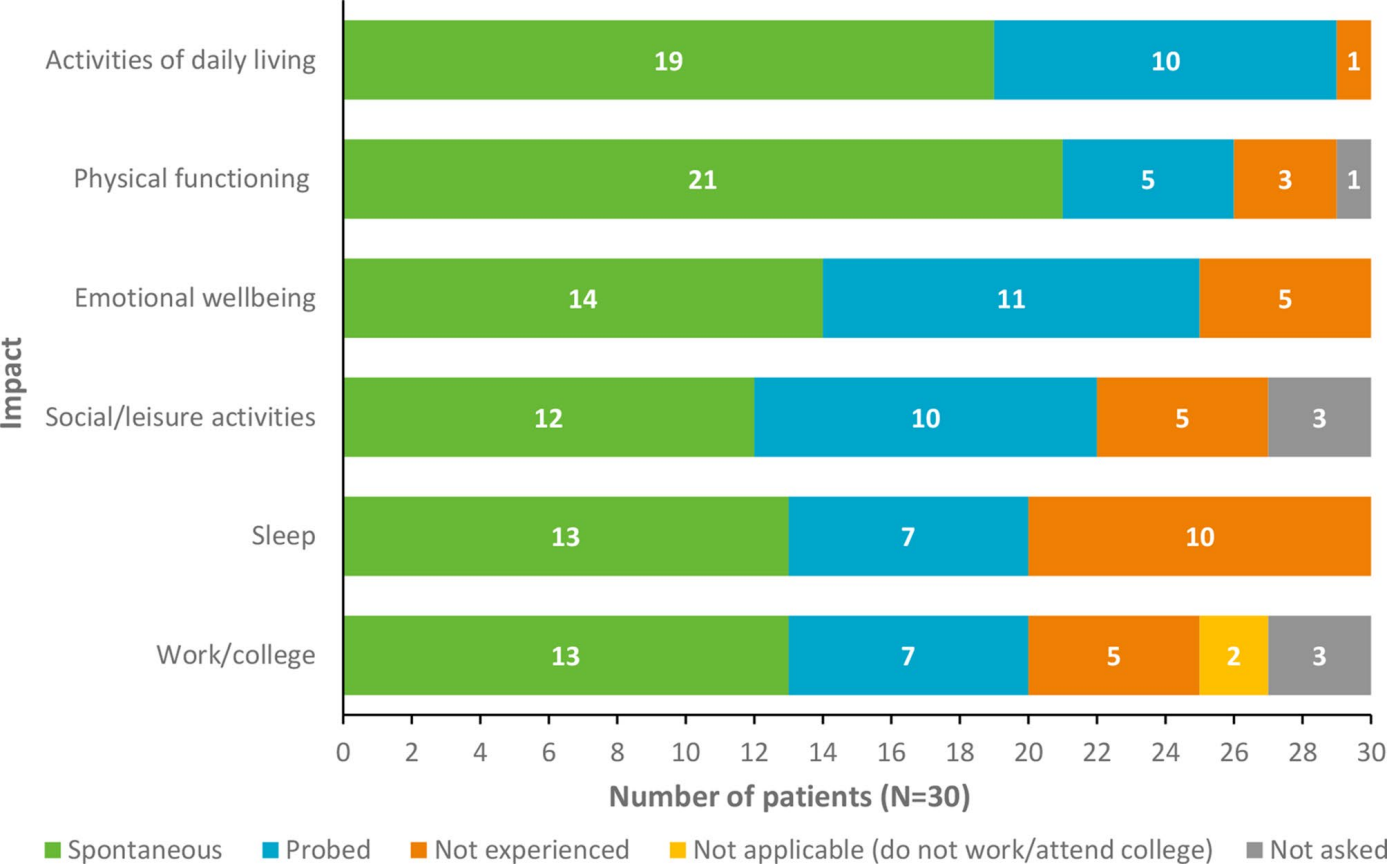
Participant-reported impacts on HRQoL (*N*=30). **Note**: Counts are not intended to quantify prevalence, but instead aim to illustrate the relevance and salience of reported concepts

Participants reported the impact of their Long COVID/PASC on HRQoL (Fig. 3). Impacts on participants’ activities of daily living (ADLs; *n* = 29/30; 97%), physical functioning (*n* = 26/29; 90%) and emotional wellbeing (*n* = 25/30; 83%) were most frequently reported when accounting for spontaneously and probed responses.

Conceptual saturation was achieved for all but one concept (vomiting), which was spontaneously elicited by one participant in the final set of interviews; however, all relevant concepts were elicited in the first three sets of interviews and sample size was sufficient.

#### Sub-group analyses

For exploratory purposes, data relating to the symptom experience of high-risk versus low-risk of severe COVID-19 sub-groups were compared. Overall, no notable differences in the severity, bothersomeness, or types of signs and symptoms reported were observed. Further results of these sub-group analyses for the most commonly reported symptoms can be found in the supplemental materials.

### Clinician CE interviews

Findings largely corroborated participant interview findings, thus aligned with concepts chosen for assessment within the Long COVID/PASC PRO instrument; 28 signs and symptoms were reported by clinicians (*N* = 3), however the most frequent were those associated with brain fog/neurological symptoms (e.g., difficulty concentrating, memory loss; *n* = 3/3), post-exertional malaise (PEM; *n* = 2/3), and gastrointestinal symptoms (e.g., diarrhea and nausea; *n* = 2/3) which was not reflected in the participant interviews. Clinicians also described impacts of Long COVID/PASC that patients report, including, impacts on work and school (*n* = 3/3), and ADLs (*n* = 3/3) with particular mention of difficulties completing daily chores, which they attributed to general tiredness and PEM. Finally, when providing feedback on the conceptual model, all clinicians agreed that it was comprehensive of the symptom experience and included all relevant and commonly reported signs and symptoms of Long COVID/PASC.

#### Participant cognitive debriefing

All participants understood and interpreted the instruction for completing the Long COVID/PASC PRO instrument as intended.

Overall, most participants demonstrated understanding of all the items retained in the final version of the Long COVID/PASC PRO instrument (90%-100%). Instances of misinterpretation were not greater than *n* = 2 per item.

**Fig. 4:**
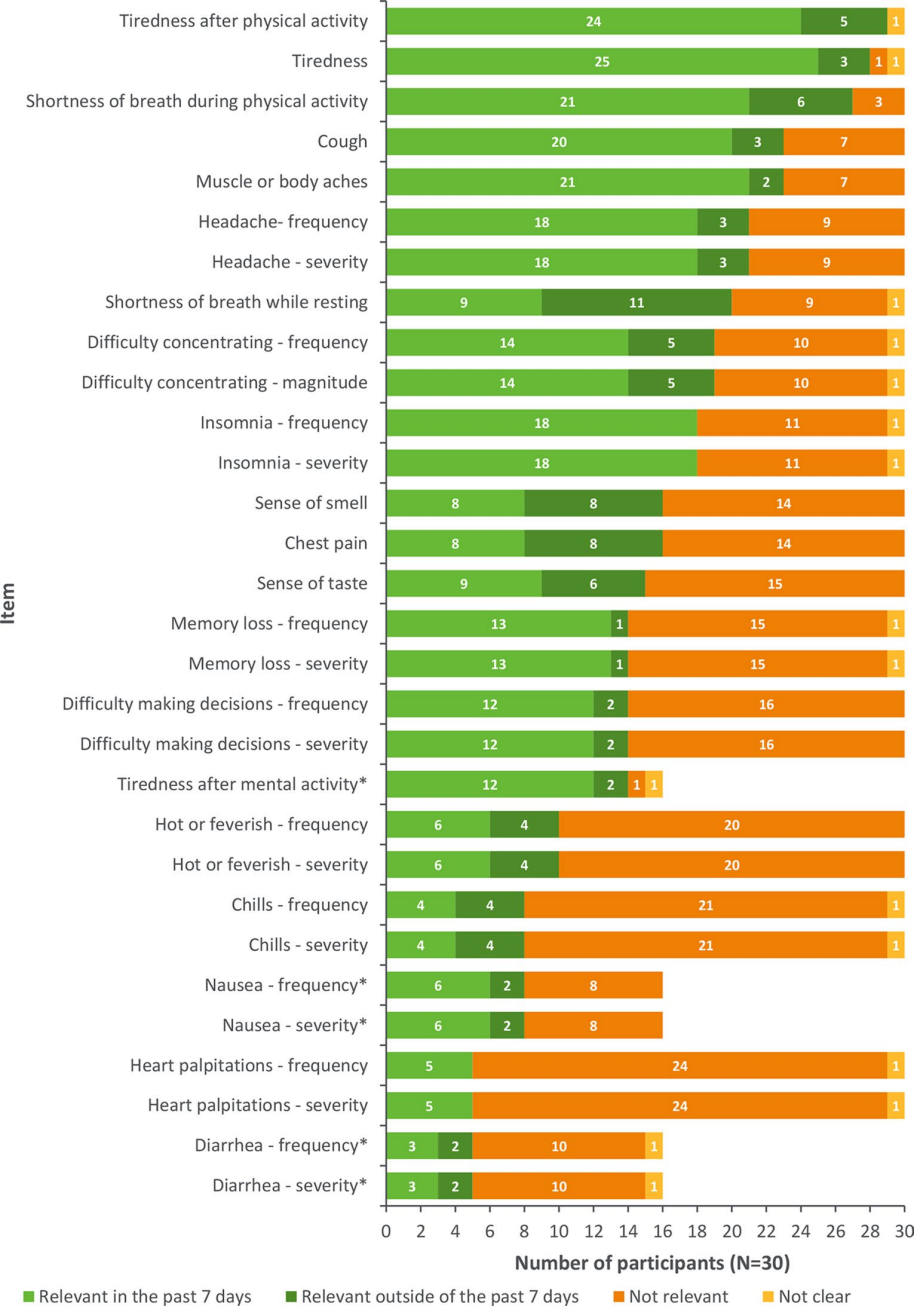
Summary of item relevance for the Long COVID/PASC symptom instrument *Only debriefed in round 2 (*N*=16)

The majority of items tested across both rounds were deemed relevant to the participant experience, both within and outside of the recall period; most relevant items included tiredness (*n* = 25; 83%), tiredness after physical activity (*n* = 24; 80%), tiredness after mental activity (*n* = 12; 75% [round 2 only]), muscle or body aches (*n* = 21; 70%), and shortness of breath during physical activity (*n* = 21; 70%) (Fig. 4).

A 4-point VRS was employed for all items except the change in smell and change in taste items which used a 3-point VRS, and the tiredness after mental activity, tiredness after physical activity, and shortness of breath after physical activity, which employed a 5-point VRS. The response options were deemed appropriate by ≥ 25 participants (≥ 83%) for most items, capturing the full range of potential severity/frequency for each concept.

Most participants (*n* ≥ 26; 87%) found the 7-day recall period appropriate across all items assessed during round 1 and round 2, thus was not modified.

#### Item-level meaningful change

In round 2, participants were asked which response option they would select if they had experienced a change in their symptom (depending on the item) that would be meaningful to them. Up to six participants discussed meaningful change for each item, and only on items which they had deemed relevant to their experience, and where there was time to discuss this during the interview. A one-point change (improvement) was considered meaningful across all items except items assessing tiredness after mental activity, diarrhea (severity and frequency), nausea (frequency), difficulty making decisions (severity), heart palpitations (severity), chest pain, chills (frequency and severity), and fever (severity). For these items, a change of two or three points was deemed meaningful; however, sample sizes were very low for each item (*n* ≤ 2).

#### Overview of item modifications

Item selection and modifications for the Long COVID/PASC PRO instrument were based on qualitative evidence for frequency of report in CE, item relevance in CD, participant preference for item wording format (severity or frequency), participant comprehension, clinical relevance and relevance within published literature. Revisions were made iteratively following each round of interviews. For example, the tiredness after mental activity, nausea, and diarrhea items were included following round 1 based on clinician feedback and the concept being well supported in the literature [13, 29].

Frequency and severity versions of certain items were included in the interviews to gauge participant understanding and preference. Following round 2 of interviews, the frequency items for nausea, diarrhea, insomnia, and heart palpitations were retained due to a higher proportion of the sample preferring this format, while the frequency version of the memory loss item was removed due to participants (*n* = 5) reporting issues with the understanding of this format. The severity version of the headaches item was retained as this aligned with the International Classification of Headache Disorders, third edition (ICHD-3) [30], as well as best practice in PRO measurement of pain [31].

Three concepts were removed from the Long COVID/PASC PRO instrument following the second round of interviews due to potential redundancy with other items and lack of relevance as reported by most of the sample. Finally, an abdominal pain item was added following a series of reports published by the FDA’s PFDD initiative [32], and mention of abdominal pain during CE interviews.

#### Final Long COVID/PASC PRO instrument

**Fig. 5:**
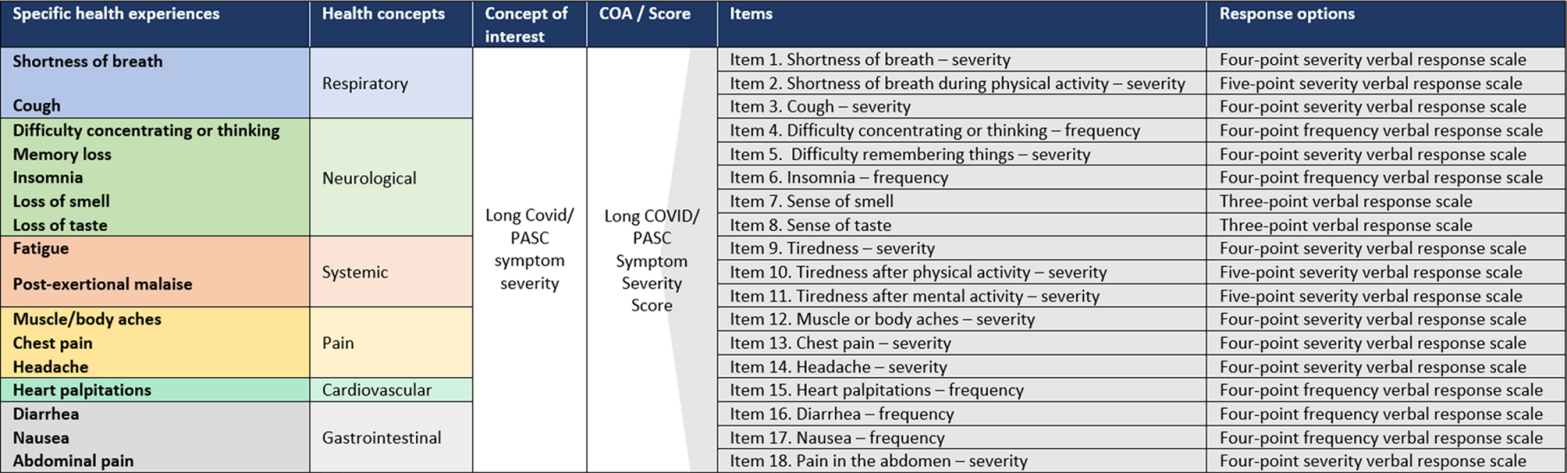
Conceptual framework for the Long COVID/PASC symptom PRO instrument

The preliminary 17-item Long COVID PRO instrument was based on the evidence review and initial conceptual model. Following two rounds of combined CE and CD interviews, a total of 18 items assessing the severity and frequency of core signs and symptoms across six domains and one instruction were retained in the final version. Three items assess respiratory symptoms, five items assess neurological symptoms, three items assess systemic symptoms, three items assess pain symptoms, one item assesses a cardiovascular symptom, and three items assess gastrointestinal symptoms. The instrument is designed to be administered once a week, and respondents are instructed to report their experience during the past 7 days using a severity 5-point (3 items), severity 4-point (8 items), frequency 4-point (4 items) and severity 3-point (2 items) VRS. A conceptual framework (Fig. 5) illustrates sign/symptom experiences and how they are assessed by the Long COVID/PASC PRO instrument.

## Discussion

Patients should be involved in developing instruments for use in clinical trials, ensuring the measures accurately reflect their experiences. An initial evidence review highlighted that no PRO instruments had been developed to assess Long COVID/PASC, according to regulatory or best practice standards, that had demonstrated content and psychometric validity. Existing instruments such as the Modified COVID-19 Yorkshire Rehabilitation Scale (C19-YRSm) [33], Symptom Burden Questionnaire-Long COVID (SBQ-LC) [34], and the COVID-19 signs and symptoms diary [20] were not considered suitable for patients to complete in a clinical trial. A recently developed instrument, the symptoms evolution of long COVID‑19 (SE-LC19) [35], was developed using similar methodology to the present study; however, many items were retained regardless of relevance and potential conceptual overlap. As this measure contains 40-items it may be considered burdensome for patients within a clinical trial with extended follow-up.

Conceptual comprehensiveness of the Long COVID/PASC PRO instrument was evaluated via qualitative interviews in alignment with best practice [16, 19]. The CE interviews demonstrated that participants experienced a range of Long COVID/PASC signs and symptoms, which broadly reflect the most commonly identified signs and symptoms in the published literature [12, 13, 36]. The most common spontaneously reported symptoms were general tiredness, shortness of breath, headache and cough. Tiredness after physical activity and muscle and body aches were also frequently reported once probed upon – this reflects that certain signs and symptoms may not be attributed to Long COVID/PASC until explicitly mentioned to the patient. Clinician interview findings largely corroborated what was elicited in the participant interviews; brain fog/neurological symptoms and tiredness were identified as the most frequent spontaneously-reported symptoms. Gastrointestinal symptoms were commonly reported by clinicians but were infrequently spontaneously-reported by participants. This discrepancy may reflect participants’ lack of attribution of gastrointestinal symptoms to Long COVID/PASC. Furthermore, the smaller clinician interview sample size limits the ability to draw definitive comparisons between the two groups; however, this highlights the importance of patient involvement when developing PRO instruments for Long COVID/PASC, given symptom experience is best known to patients.

CD findings confirmed the relevance and comprehensibility of the concepts assessed in the instrument, with the majority of items tested across both rounds being deemed relevant. While it is noted that heart palpitations, diarrhea, nausea, and abdominal pain were reported to be relevant to fewer participants in comparison to other signs and symptoms assessed by this instrument, clinical input and recent literature [13] supported their retention. Additionally, the aim of having an overly comprehensive measure in the qualitative stages of development, to be tested in larger samples quantitatively supported inclusion of these concepts. Overall, evidence generated from this study supports the conceptual comprehensiveness of the Long COVID/PASC PRO instrument and therefore its use in Long COVID/PASC.

A central tenet of content validity is ensuring that the instructions, item wording and response options are interpreted by participants as intended [37]. Overall, comprehension for every item tested across both rounds of interviews was as intended, with the item and instruction wording, response options and recall period interpreted as intended across the instrument. Participant interview data supported the use of a 7-day recall, with the majority of signs and symptoms commonly described as being experienced weekly and participants broadly reporting no issue in recalling their symptom experience over 7 days. Response options were deemed appropriate by most participants for the majority of items in CD interviews, capturing the full range of potential severity/frequency for each concept.

### Generalizability of findings

Purposeful sampling methods were used to capture representation within the Long COVID/PASC population, and ensured that the sample represented patients with various characteristics.

Targets implemented to promote demographic and clinical diversity of the recruited participant sample were also met, providing further confidence of representation. The range in educational status across the sample is also a strength, given it is important to evaluate the understanding of item wording in participants with different educational/literacy backgrounds.

Although there is a large amount of heterogeneity among participants’ experiences of Long COVID/PASC, and some aspects may not have been captured in the data, all but one symptom concept emerged spontaneously by the fourth group of interviews, indicating that conceptual saturation was achieved.

### Limitations

Exploratory sub-group analyses were performed, but no conclusions can be drawn due to the small sample sizes of the groups of interest. Additionally, due to the heterogeneity of Long COVID/PASC, the assessment of some aspects of the patient experience may be omitted from the Long COVID/PASC PRO instrument, or any other PRO instruments of Long COVID/PASC. Nevertheless, the CD interview findings support that the Long COVID/PASC PRO instrument assesses the most salient signs and symptoms experienced by participants.

### Conclusion

The present study has provided insights into the patient experience of Long COVID/PASC, eliciting the signs and symptoms reported to be most frequent and salient in terms of severity and bother, as well as the HRQoL impacts of the condition. Moreover, the Long COVID/PASC PRO instrument has been developed in line with FDA regulatory standards and demonstrated content validity in a Long COVID/PASC population. To further support the development of the Long COVID/PASC PRO instrument, next steps could include an evaluation of its quantitative measurement properties in a Long COVID/PASC population.

## Supplementary Information

Below is the link to the electronic supplementary material.

Supplementary Material 1

## Data Availability

The datasets generated and/or analyzed during the current study are not publicly available to protect participant confidentiality.

## Abbreviations

ADL: Activities of Daily Living
CD: Cognitive Debriefing
CDC: Centers for Disease Control and Prevention
CFQ: Cognitive Failures Questionnaire
CE: Concept Elicitation
EMA: European Medicines Agency
FDA: Food and Drug Administration
HRQoL: Health-related quality of life
ICHD-3: The International Classification of Headache Disorders Third Edition
NAAT: Nucleic Acid Amplification Test
NICE: National Institute for Health and Care Excellence
PASC: Post-acute sequelae of SARS-CoV-2 infection
PC-COS: Post-COVID Core Outcome Set
PEM: Post exertional malaise
PFDD: Patient-Focused Drug Development series
PRO: Patient-Reported Outcome
SARS-CoV-2: Severe acute respiratory syndrome coronavirus 2
USA: United States of America
VRS: Verbal Response Scale
WCG IRB: Western Copernicus Group Independent Review Board
WHO: World Health Organization
