# Supplementary material for: Development and content validation of the Long COVID/ post-acute sequelae of COVID-19 (PASC) patient-reported outcome (PRO) instrument
Source: J Patient Rep Outcomes. 2025 Aug 20;9:105. doi: 10.1186/s41687-025-00942-w (PMC12367622; doi:10.1186/s41687-025-00942-w)
Supplement: Supplementary file 1 — Supplementary Material 1 [file 41687_2025_942_MOESM1_ESM.docx]

# Supplementary materials


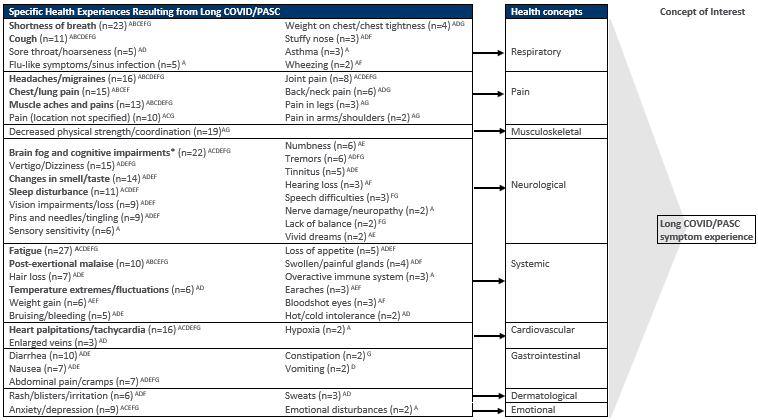


Supplementary Figure 1. Conceptual model of Long COVID/PASC derived from the evidence review

*A: Qualitative literature review search via Medline and PsycInfo databases, and additional searches via Google, Long COVID websites, and data sources from medical organizations/patient advocacy groups (n=14 articles; n=6 patient stories, see below for references); B: FDA COVID-19 signs and symptoms list; C: FDA PFDD Long COVID meeting; D: Long Covid Symptom and Impact Tools; E: Modified COVID-19 Yorkshire Rehabilitation Scale (C19-YRSm); F: Symptom Burden Questionnaire- Long COVID (SBQ- LC); G: Post-COVID Condition Core Outcomes (PC-COS); * Includes impaired concentration, impaired memory, confusion, trouble processing or speaking correctly. Bold indicates that concept selected for inclusion in the first draft of the Long COVID/PASC PRO instrument. N=number of evidence sources where concept was identified. Total number of evidence sources=26.*

| Supplementary Table 1. Qualitative literature review article references | |
| --- | --- |
| Qualitative literature review material | References |
| Articles (n=14) | - Kingstone T, Taylor AK, O'Donnell CA, Atherton H, Blane DN, Chew-Graham CA. Finding the'right'GP: a qualitative study of the experiences of people with long-COVID. BJGP open. 2020 Dec 1;4(5). - Shelley J, Hudson J, Mackintosh KA, Saynor ZL, Duckers J, Lewis KE, Davies GA, Berg RM, McNarry MA. ‘I Live a Kind of Shadow Life’: individual experiences of COVID-19 recovery and the impact on physical activity levels. International journal of environmental research and public health. 2021 Oct 29;18(21):11417. - Humphreys H, Kilby L, Kudiersky N, Copeland R. Long COVID and the role of physical activity: a qualitative study. BMJ open. 2021 Mar 1;11(3):e047632. - Bilgin A, Kesik G, Ozdemir L. ‘The body seems to have no life’: The experiences and perceptions of fatigue among patients after COVID‐19. Journal of Clinical Nursing. 2024 Jan;33(1):126-36. - Rushforth A, Ladds E, Wieringa S, Taylor S, Husain L, Greenhalgh T. Long Covid–the illness narratives. Social science & medicine. 2021 Oct 1;286:114326. - Burton A, Aughterson H, Fancourt D, Philip KE. Factors shaping the mental health and well-being of people experiencing persistent COVID-19 symptoms or ‘long COVID’: qualitative study. BJPsych open. 2022 Mar;8(2):e72. - Aghaei A, Zhang R, Taylor S, Tam CC, Yang CH, Li X, Qiao S. Social life of females with persistent COVID-19 symptoms: a qualitative study. International Journal of Environmental Research and Public Health. 2022 Jul 26;19(15):9076. - Schiavi M, Fugazzaro S, Bertolini A, Denti M, Mainini C, Accogli MA, Bedogni G, Ghizzoni D, Esseroukh O, Gualdi C, Costi S. “Like before, but not exactly”: the Qualy-REACT qualitative inquiry into the lived experience of long COVID. BMC Public Health. 2022 Mar 28;22(1):599. - Moretti C, Collaro C, Terzoni C, Colucci G, Copelli M, Sarli L, Artioli G. Dealing with uncertainty. A qualitative study on the illness’ experience in patients with long-COVID in Italy. Acta Bio Medica: Atenei Parmensis. 2022 Dec 16;93(6):e2022349. - Chasco EE, Dukes K, Jones D, Comellas AP, Hoffman RM, Garg A. Brain fog and fatigue following COVID-19 infection: an exploratory study of patient experiences of long COVID. International journal of environmental research and public health. 2022 Nov 23;19(23):15499. - Almgren J, Löfström E, Malmborg JS, Nygren J, Undén J, Larsson I. Patients’ health experiences of post COVID-19 condition—A qualitative study. International Journal of Environmental Research and Public Health. 2022 Oct 27;19(21):13980. |
|  | - Wurz A, Culos-Reed SN, Franklin K, DeMars J, Wrightson JG, Twomey R. " I feel like my body is broken": exploring the experiences of people living with long COVID. Quality of Life Research. 2022 Dec;31(12):3339-54. - Loft MI, Foged EM, Koreska M. An unexpected journey: the lived experiences of patients with long-term cognitive sequelae after recovering from COVID-19. Qualitative Health Research. 2022 Jul;32(8-9):1356-69. - Pearson M, Singh P, Bartel H, Crawford P, Allsopp G. Creative Long Covid: A qualitative exploration of the experience of Long Covid through the medium of creative narratives. Health Expectations. 2022 Dec;25(6):2950-9. |
| Patient stories (n=6) | - Long COVID Ireland – Patient stories of n=17 individuals with Long COVID/PASC - Long COVID SOS – Patient stories of n=5 individuals with Long COVID/PASC - British Heart Foundation – Patient stories of n=3 individuals with Long COVID/PASC - World Health Organization – Patient stories of n=6 individuals with Long COVID/PASC - Hounslow and Richmond Community Healthcare NHS Trust – YouTube interview with a patient diagnosed with Long COVID/PASC - Alberta Health Services – Three YouTube interviews with patients diagnosed with Long COVID/PASC |


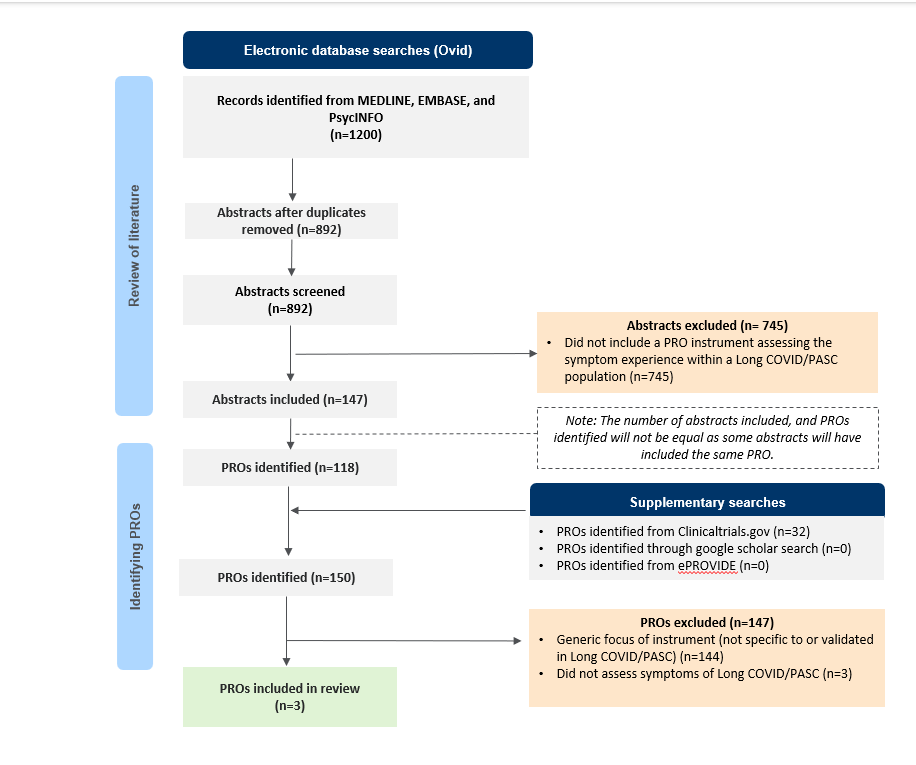


Supplementary Figure 2. Overview of the Long COVID/PASC instrument review

| **Supplementary Table 2. Topline overview of the Long COVID/PASC instrument review** | | | |
| --- | --- | --- | --- |
| **PRO** | **Evidence of face validity** | **Evidence of content validity** | **Review of measurement properties** |
| **Modified COVID-19 Yorkshire Rehabilitation Scale (C19-YRSm)** | - **Good face validity** evidence related to the concepts assessed by the measure, and scoring method. - **Poor face validity** evidence related to the item and/or instruction wording, response options, and recall period. | - **Good content validity** evidence related to stakeholder input in development. - **No evidence** related to patient input in development, and patient research to evaluate understanding. | - **Good evidence identified** for item response theory/Rasch analysis. - **No evidence** identified for item-level analyses, factor analyses, construct validity, reliability, ability to detect change or score interpretation. |
| **Symptom Burden Questionnaire- Long COVID (SBQ- LC)** | - **Good face validity** evidence related to item and/or instruction wording, response options, recall period and scoring method. - **Poor face validity** evidence related to the concepts assessed by the measure. | - **Good content validity** evidence related to stakeholder input in development, and patient research to evaluate understanding. - **No evidence** related to patient input in development. | - **Good evidence identified** for quality of completion, item response theory/Rasch analysis. - **Poor evidence identified** for response distribution (floor or ceiling effects), and mixed evidence for internal consistency. - **No evidence** identified for factor analysis, construct validity, test-retest reliability, ability to detect change or score interpretation. |
| **Long Covid Symptom and Impact Tools** | - **Good face validity** evidence for item and/or instruction wording and scoring method. - **Poor face validity** evidence related to concepts assessed by the measure, response options and recall period. | - **Good content validity** evidence related to patient input in development. - **No evidence** of input from stakeholders in development or patient research to evaluate understanding. | - **Good evidence identified** for convergent validity and test-retest reliability. - **No evidence** identified for item-level analyses, item response theory/Rasch analysis, known-groups validity, predictive validity, internal consistency, ability to detect change and score interpretation. |

Supplementary Table 3. Patient eligibility criteria

| **Inclusion criteria** | **Exclusion criteria** |
| --- | --- |
| Males and females ≥18 years of age who meet WHO criteria* for confirmed COVID-19 infection. *[*positive Nucleic Acid Amplification Test (NAAT) or Rapid Antigen Test].* | Participant diagnosed with COVID-19 or any other respiratory infection within 30 days from study enrollment. |
| Participant is currently experiencing symptoms due to COVID-19. The symptoms should have been present on day 30 after their diagnosis OR started after day 30 and have lasted for at least 60 days. The symptoms may be persistent, recurrent or may have developed after the initial infection. | Participant’s Long COVID/PASC symptoms started before February 2022 (pre-Omicron era). |
| Participant has experienced at least 2 Long COVID/PASC symptoms of moderate or severe intensity. | Participant has other current or previous medical condition(s), or concomitant therapy, that would compromise participant’s safety or compliance with the study protocol. |
| Participant is willing to report all COVID-19 vaccinations. | Participant has other current or previous medical condition(s), or concomitant therapy, that have similar symptom presentations to Long COVID/PASC, therefore posing significant risk of confounding interpretation of study results (including, but not exclusively, rheumatoid arthritis, fibromyalgia, chronic fatigue syndrome, rheumatic diseases, known cognitive dysfunction, pulmonary disease associated with dyspnea [COPD and poorly controlled asthma]). |
| Participant is able to read, write and fully understand the English language. | Participant is currently enrolled in, or has discontinued participation in, a clinical trial involving any investigational drug or device within the past 30 days |
| Participant resides in the US. | Participant is unable to provide informed consent |
| Participant is willing, and physically and psychologically able, to take part in a 90-minute interview to discuss their experience of Long COVID/PASC symptoms and provide feedback on a questionnaire. |  |
| Evidence of a personally signed and dated informed consent document indicating that the Participant (or a legally acceptable representative) has been informed of all pertinent aspects of the study. |  |

| **Supplementary Table 4. Patient clinical characteristics** | | | | |
| --- | --- | --- | --- | --- |
| Clinical characteristics | | Round 1 (n=14) | Round 2 (n=16) | Total (N=30) |
| Test used to confirm COVID-19 infection, n (%) | Rapid antigen test | 10 | 11 | 21 (70%) |
|  | PCR test result | 4 | 4 | 8 (27%) |
|  | Unknown | 0 | 1 | 1 (3%) |
| Time (months) from index infection of COVID-19 to the diagnosis of Long COVID/PASC | Mean (SD) | 3 (3.0) | 3 (2.0) | 2.8 (2.4) |
|  | Median | 2 | 3 | 2 |
|  | Range | 1-13 | 1-8 | 1-13 |
| Time (days) diagnosed with Long COVID/PASC symptoms* | Mean (SD) | 94.4 (13.9) | 90.7 (11.6) | 92.4 (12.7) |
|  | Median | 99 | 90 | 90 |
|  | Range | 78-120 | 70-120 | 70-120 |
| Number of times infected with COVID-19, n (%) | One | 8 | 11 | 19 (63%) |
|  | Two | 6 | 5 | 11 (37%) |
| Number of vaccination(s) received in total, n (%) | Completed primary series (two doses) | 5 | 7 | 12 (40%) |
|  | Up to date (three doses) | 6 | 2 | 8 (27%) |
|  | At least one dose | 1 | 4 | 5 (17%) |
|  | Unvaccinated | 2 | 3 | 5 (17%) |
| Hospitalization (for COVID-19) status, n (%) | Not hospitalized | 13 | 16 | 29 (97%) |
|  | Hospitalized | 1 | 0 | 1 (3%) |
| COVID-19 vaccination(s) received, n (%) | Pfizer – BioNTech | 10 | 11 | 21 (70%) |
|  | Unvaccinated | 2 | 3 | 5 (17%) |
|  | Moderna | 1 | 2 | 3 (10%) |
|  | Janssen/JnJ vaccine | 1 | 0 | 1 (3%) |
| Treatment received for COVID-19**, n (%) | No treatment received | 13 | 16 | 29 (97%) |
|  | Steroid pack treatment | 1 | 0 | 1 (3%) |
| Timeframe of COVID-19 vaccination in relation to the experience of Long COVID/PASC symptoms, n (%) | Received vaccinations before symptoms | 9 | 11 | 20 (67%) |
|  | Received vaccinations after symptoms | 3 | 2 | 5 (17%) |
|  | Unvaccinated | 2 | 3 | 5 (17%) |
| Diagnosis of current or previous medical conditions (co-morbidities)*** ^†^, n (%) | Gastroesophageal reflux disease | 0 | 3 | 3 (10%) |
|  | Anxiety | 0 | 3 | 3 (10%) |
|  | Diabetes | 1 | 1 | 2 (7%) |
|  | Depression | 1 | 1 | 2 (7%) |
|  | Diabetes Mellitus | 1 | 1 | 2 (7%) |
|  | Arthritis in knee | 1 | 0 | 1 (3%) |
|  | Myocardial Infarction | 1 | 0 | 1 (3%) |
|  | High blood pressure | 1 | 0 | 1 (3%) |
|  | Atopic dermatitis | 1 | 0 | 1 (3%) |
|  | Hypertension | 1 | 0 | 1 (3%) |
|  | Cholesterol | 1 | 0 | 1 (3%) |
|  | Lipids | 0 | 1 | 1 (3%) |
|  | Asthma | 0 | 1 | 1 (3%) |
| Risk classification of experiencing severe outcomes of COVID-19^‡^ | High-risk | 8 | 10 | 18 (60%) |
|  | Low-risk | 6 | 6 | 12 (40%) |
| **Patients may have been experiencing symptoms at the time of the interview.*  ***Treatment was self-reported by the patient.*  ****Some patients reported more than one current or previous medical condition.*  *^†^Co-morbidities were not diagnosed by the research team as part of the current study. This was only reported via a clinician-completed form.*  *^‡^As defined by evidence-based resources published by the Centers for Disease Control and Prevention (CDC)* | | | | |

| **Supplementary Table 5. Summary of sub-group analyses in high-risk vs low-risk participants** | | | | | | |
| --- | --- | --- | --- | --- | --- | --- |
| **Symptom reported** | **Symptom frequency N (%)** | | **Mean severity rating (0-10)** | | **Mean bothersome rating (0-10)** | |
|  | High risk (n=18) | Low risk (n=12) | High risk (n=18) | Low risk (n=12) | High risk (n=18) | Low risk (n=12) |
| **Tiredness after physical activity** | n=18 (100%) | n=11 (92%) | 5.4 | 6.1 | 5.5 | 6.2 |
| **Tiredness** | n=17 (94%) | n=11 (92%) | 5.2 | 6.0 | 6.9 | 6.8 |
| **Shortness of breath at rest** | n=15 (84%) | n=9 (75%) | 5.1 | 5.3 | 6.6 | 6.5 |
| **Cough** | n=15 (84%) | n=8 (67%) | 4.5 | 4.2 | 5.0 | 5.6 |
| **Muscle and body aches** | n=15 (84%) | n=8 (67%) | 6.1 | 6.3 | 6.0 | 6.5 |
| **Headaches** | n=11 (61%) | n=10 (83%) | 5.8 | 7.5 | 6.1 | 7.1 |
| **Shortness of breath after physical activity** | n=12 (67%) | n=7 (58%) | 5.8 | N/A - Not reported | 5.5 | N/A - Not reported |
| **Difficulty concentrating or thinking** | n=10 (56%) | n=9 (75%) | 5.7 | 6.1 | 6.9 | 6.8 |
| **Insomnia** | n=8 (44%) | n=10 (83%) | 6.2 | 6.7 | 7.6 | 7.5 |
| *Note: Severity was rated between 0 and 10, where 0 was not at all severe and 10 was the worst severity they could imagine. Similarly, bothersome was rated between 0 and 10, where 0 was not bothersome at all and 10 was the most bothersome they could imagine.*  *The high-risk group was defined as participants who were 50+ years old, and/or had a comorbid condition (i.e., asthma, diabetes, heart conditions), and/or were unvaccinated, in line with CDC resources and classification. Remaining* participants *were defined as low-risk.*  *Not all* participants *were asked to report the severity and bothersome score due to time constraints; therefore, the averages were only calculated out of those who were asked.* | | | | | | |

| **Supplementary Table 6. Clinician characteristics** | | | | | | | |
| --- | --- | --- | --- | --- | --- | --- | --- |
| **ID** | **Location** | **Clinical specialty** | **Time as a practicing clinician** | **Experience with Long COVID/PASC patients** | **Work settings** | **Involvement in Long COVID/PASC and COVID-19 scientific papers and clinical trials** |  |
| **CL-01** | Austin, TX | Internal medicine | 8 years | - Consulting and managing patients with Long COVID/PASC for 2-3 years. - Sees 50 adult patients with Long COVID/PASC per month. | - Clinics. - Hospital-based care. - University and/or college affiliated. | - Two papers published in Long COVID/PASC. - Six papers published in COVID-19. - Previously worked/ currently working in one clinical trial for Long COVID/PASC. - Previously worked/ currently working in one clinical trial for COVID-19. |  |
| **CL-02** | San Francisco, CA | Infectious diseases | 10 years | - Consulting and managing patients with Long COVID/PASC for 3+ years. - Sees 50 adult patients with Long COVID/PASC per month. | - Clinics. - Hospital-based care. - University and/or college affiliated. - Research clinics. | - 25 papers published in Long COVID/PASC. - Five papers published in COVID-19. - Previously worked/ currently working in five clinical trials for Long COVID/PASC. |  |
| **CL-03** | East Haven, CT | Pulmonary and critical care | 8 years | - Consulting and managing patients with Long COVID/PASC for 3+ years. - Sees 20 adult patients with Long COVID/PASC per month. | - University or college affiliated. | - Four papers published in Long COVID/PASC. - Two papers published in COVID-19. |  |
